# Supplementary material for: S. aureus alpha-toxin monomer binding and heptamer formation in host cell membranes – Do they determine sensitivity of airway epithelial cells toward the toxin?
Source: PLoS One. 2020 May 29;15(5):e0233854. doi: 10.1371/journal.pone.0233854 (PMC7259691; doi:10.1371/journal.pone.0233854)
Supplement: S1 File — (PDF) [file pone.0233854.s001.pdf]

Supplemental Information 1, PONE -D-20-07341

S. aureus alpha-toxin monomer binding and heptamer formation in host cell membranes – Do they determine sensitivity of airway epithelial cells toward the toxin?

Nils Möller, Sabine Ziesemer, Petra Hildebrandt, Nadine Assenheimer, Uwe Völker,
Jan-Peter Hildebrandt

Original Publications about Generation and Characterization of the Cell Lines Used in these Studies

S9 Cells

Zeitlin PL, Lu L, Rhim J, Cutting G, Stetten G, Kieffer KA, et al. A cystic fibrosis bronchial epithelial cell line: immortalization by adeno-12-SV40 infection. American Journal of Respiratory Cell and Molecular Biology. 1991; 4(4):313-319. doi: 10.1165/ajrcmb/4.4.313

Flotte TR, Afione SA, Conrad C, McGrath SA, Solow R, Oka H, et al. Stable in vivo expression of the cystic fibrosis transmembrane conductance regulator with an adeno-associated virus vector. Proceedings of the National Academy of Sciences of the United States of America. 1993;90(22):10613-10617. doi: 10.1073/pnas.90.22.10613

Flotte TR, Afione SA, Solow R, Drumm ML, Markakis D, Guggino WB, et al. Expression of the cystic fibrosis transmembrane conductance regulator from a novel Adeno-associated virus promoter. Journal of Biological Chemistry. 1993;268(5):3781-3790.

Eichstaedt S, Gäbler K, Below S, Müller C, Hildebrandt J-P. Phospholipase C-activating plasma membrane receptors and calcium signaling in immortalized human

airway epithelial cells. Journal of Receptors and Signal Transduction. 2008;28:591-612. doi: 10.1016/j.ceca.2008.09.001

Richter E, Harms M, Ventz K, Gierok P, Chilukoti RK, Hildebrandt J-P, et al. A multi-omics approach identifies key hubs associated with cell type-specific responses of airway epithelial cells to staphylococcal alpha-toxin. PLoS ONE. 2015;10(3):e0122089. doi: 10.1371/journal.pone.0122089

16HBE14o- Cells

Haws C, Krouse ME, Xia Y, Gruenert DC, Wine JJ. CFTR channels in immortalized human airway cells. American Journal of Physiology 1992;263(6 Pt 1):L692-L707. doi: 10.1152/ajplung.1992.263.6.L692

Cozens AL, Yezzi MJ, Kunzelmann K, Ohrui T, Chin L, Eng K, et al. CFTR expression and chloride secretion in polarized immortal human bronchial epithelial cells. American Journal of Respiratory Cell and Molecular Biology. 1994;10(1):38-47. doi: 10.1165/ajrcmb.10.1.7507342

Kunzelmann K, Koslowsky T, Hug T, Gruenert DC, Greger R. cAMP-dependent activation of ion conductances in bronchial epithelial cells. Pflugers Archive. 1994;428(5-6):590-596. doi: 10.1007/bf00374582

Eichstaedt S, Gäbler K, Below S, Müller C, Hildebrandt J-P. Phospholipase C-activating plasma membrane receptors and calcium signaling in immortalized human airway epithelial cells. Journal of Receptors and Signal Transduction. 2008;28:591-612. doi: 10.1016/j.ceca.2008.09.001

A549 Cells

Lieber M, Smith B, Szakal A, Nelson-Rees W, Todaro G. A continuous tumor-cell line from a human lung carcinoma with properties of type II alveolar epithelial cells. International Journal of Cancer. 1976;17(1):62-70. doi: 10.1002/ijc.2910170110

Communi D, Paindavoine P, Place GA, Parmentier M, Boeynaems JM. Expression of P2Y receptors in cell lines derived from the human lung. British Journal of Pharmacology. 1999;127(2):562-568. doi: 10.1038/sj.bjp.0702560

Foster KA, Oster G, Mayer MM, Avery ML, Audus KL. Characterization of the A549 cell line as a type II pulmonary epithelial cell model for drug metabolism. Experimental Cell Research. 1998; 243(2):359-366. doi: 10.1006/excr.1998.4172

Proteomic Studies using these Cell Lines

Richter E, Harms M, Ventz K, Gierok P, Chilukoti RK, Hildebrandt J-P, et al. A multi-omics approach identifies key hubs associated with cell type-specific responses of airway epithelial cells to staphylococcal alpha-toxin. PLoS ONE. 2015;10(3):e0122089. doi: 10.1371/journal.pone.0122089

Surmann K, Simon M, Hildebrandt P, Pförtner H, Michalik S, Stentzel S, et al. A proteomic perspective of the interplay of *Staphylococcus aureus* and human alveolar epithelial cells during infection. Journal of Proteomics 2015;128:203-217. doi: 10.1016/j.jprot.2015.07.034

Palma Medina LM, Becker A-K, Michalik S, Yedavally H, Raineri EJM, Hildebrandt P, et al. Metabolic cross-talk between human bronchial epithelial cells and internalized *Staphylococcus aureus* as a driver for infection. Molecular and Cellular Proteomics 2019;18(5):892. doi: 10.1074/mcp.RA118.001138
