# Supplementary material for: S. aureus alpha-toxin monomer binding and heptamer formation in host cell membranes – Do they determine sensitivity of airway epithelial cells toward the toxin?
Source: PLoS One. 2020 May 29;15(5):e0233854. doi: 10.1371/journal.pone.0233854 (PMC7259691; doi:10.1371/journal.pone.0233854)
Supplement: S1 Fig — (PDF) [file pone.0233854.s002.pdf]

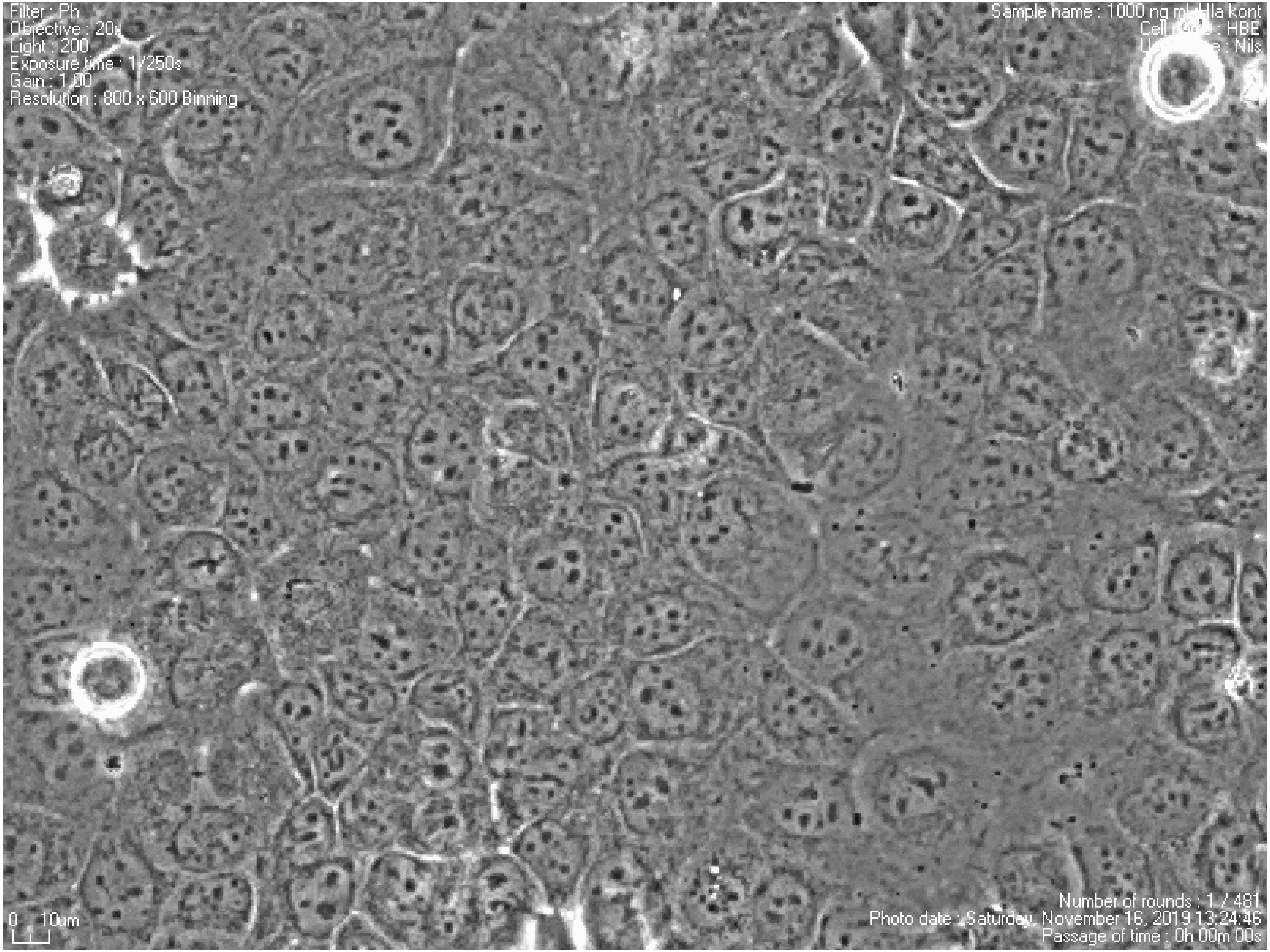
Filter : Ph
Objective : 20
Light : 200
Exposure time : 1/250s
Gain : 1.00
Resolution : 800 x 600 Binning
Sample name : 1000 ng ml Hla kont
Cell
: HBE
: Nils
0 10um
Number of rounds : 1 / 481
Photo date : Saturday, November 16, 2019 13:24:46
Passage of time : 0h 00m 00s

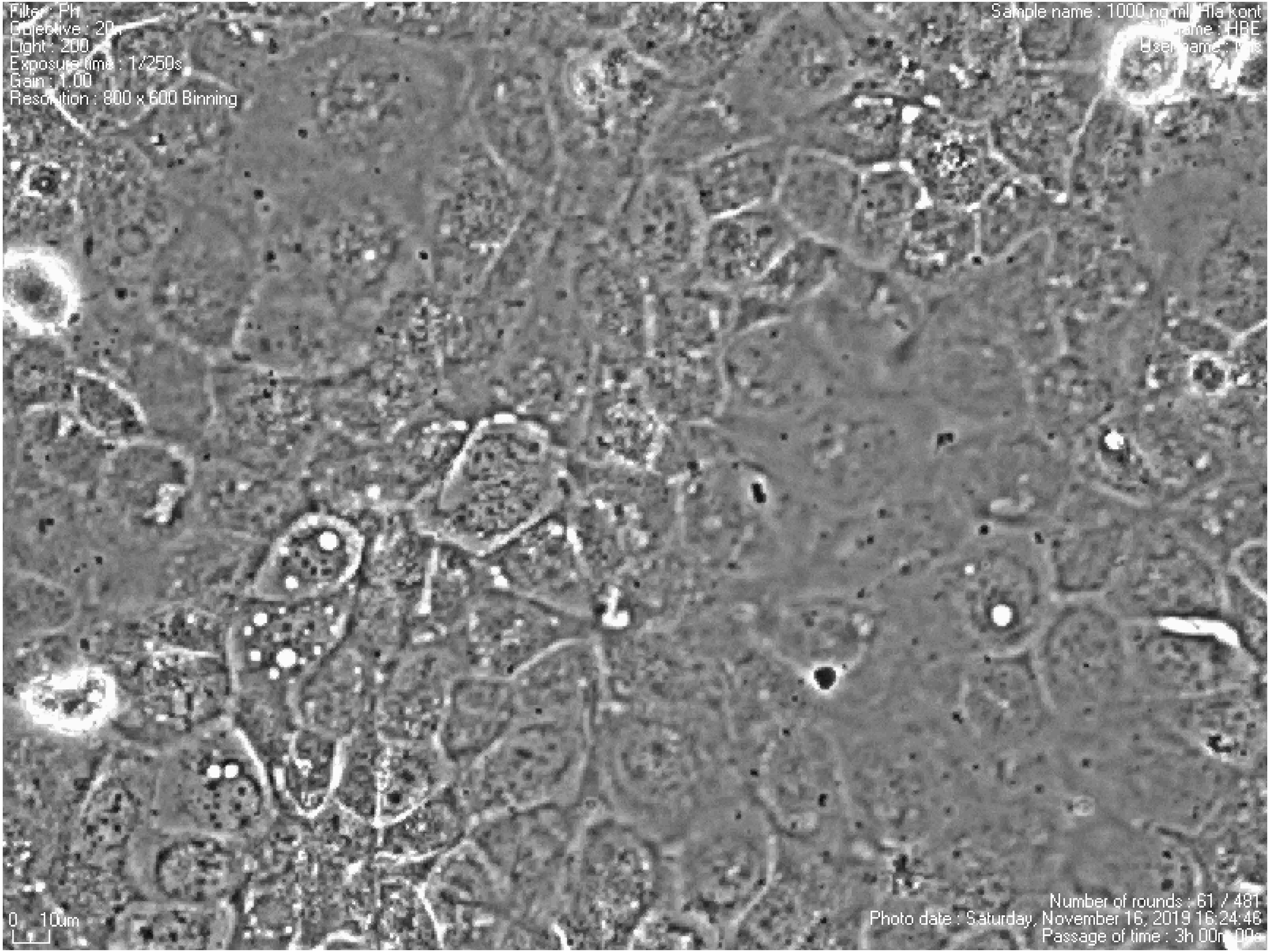
Filter : Ph
Objective : 20
Light : 200
Exposure time : 1/250s
Gain : 1.00
Resolution : 800 x 600 Binning
Sample name : 1000 ng ml Hla kont
Number of rounds : 61 / 481
Photo date : Saturday, November 16, 2019 16:24:46
Passage of time : 3h 00m 00s
0 10um

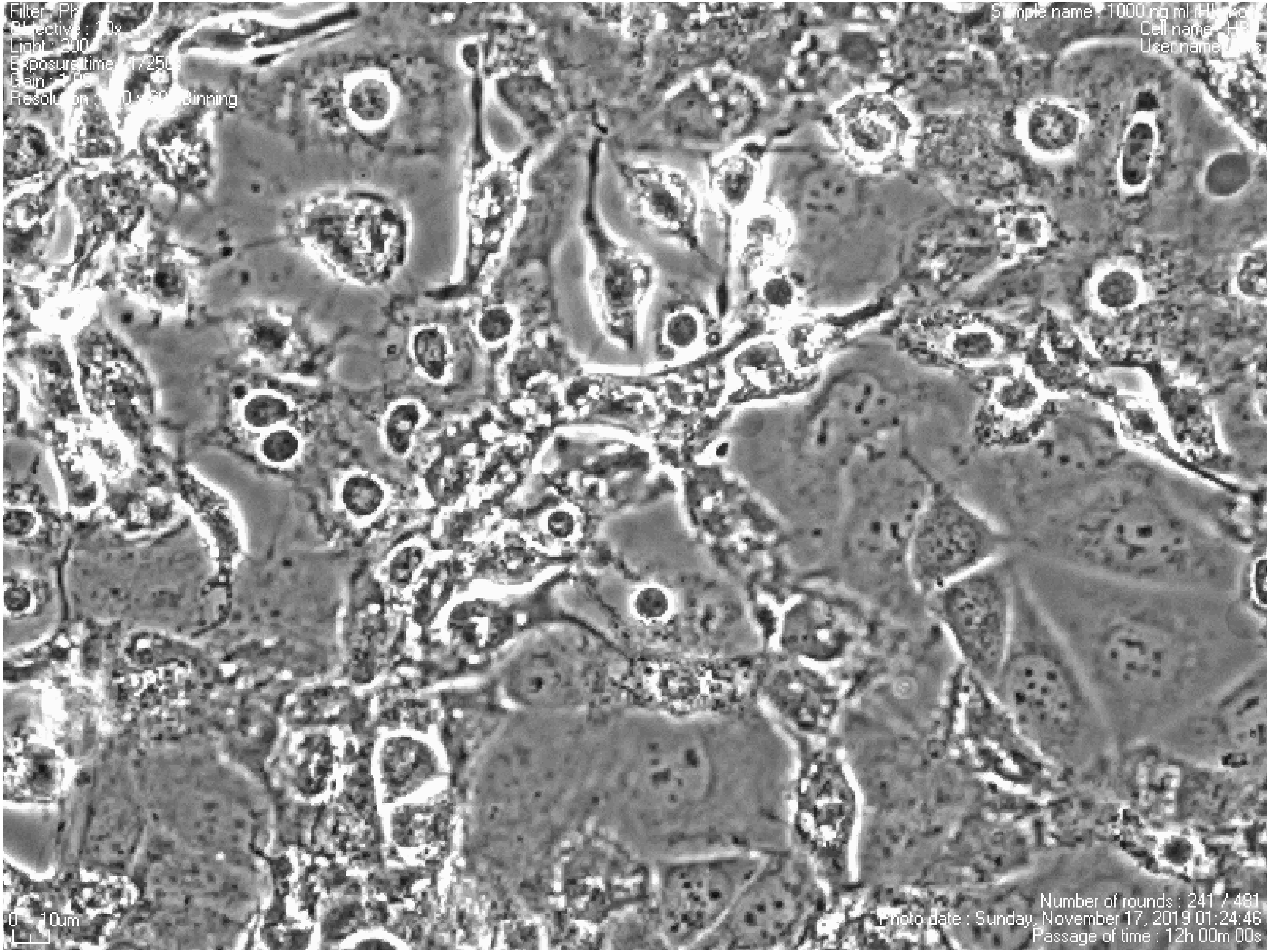
10um
Number of rounds : 241 / 481
Photo date : Sunday, November 17, 2019 01:24:46
Passage of time : 12h 00m 00s

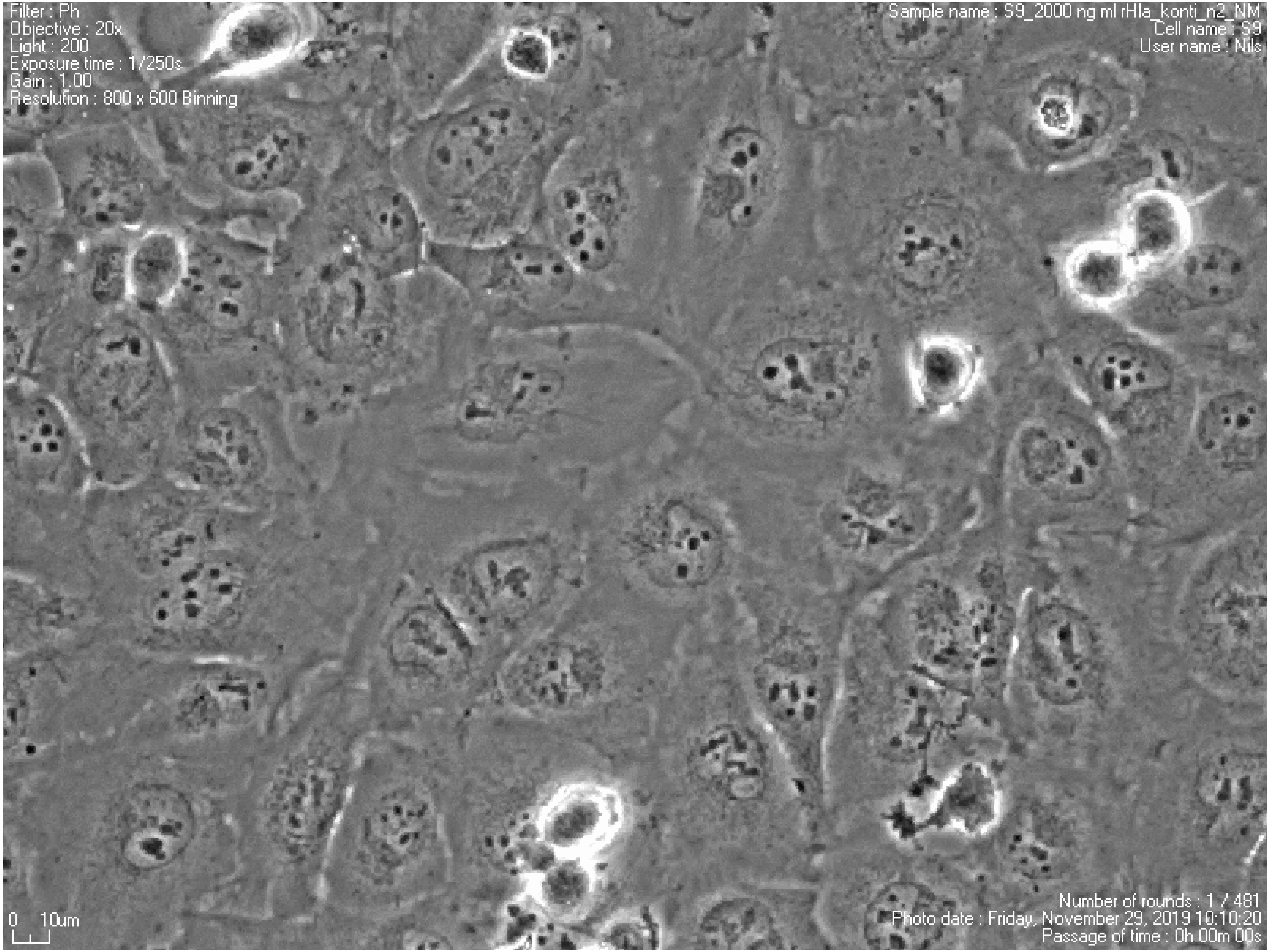
Filter : Ph
Objective : 20x
Light : 200
Exposure time : 1/250s
Gain : 1.00
Resolution : 800 x 600 Binning
Sample name : S9_2000 ng ml rHla_konti_n2_NM
Cell name : S9
User name : Nils
Number of rounds : 1 / 481
Photo date : Friday, November 29, 2019 10:10:20
Passage of time : 0h 00m 00s
0 10um

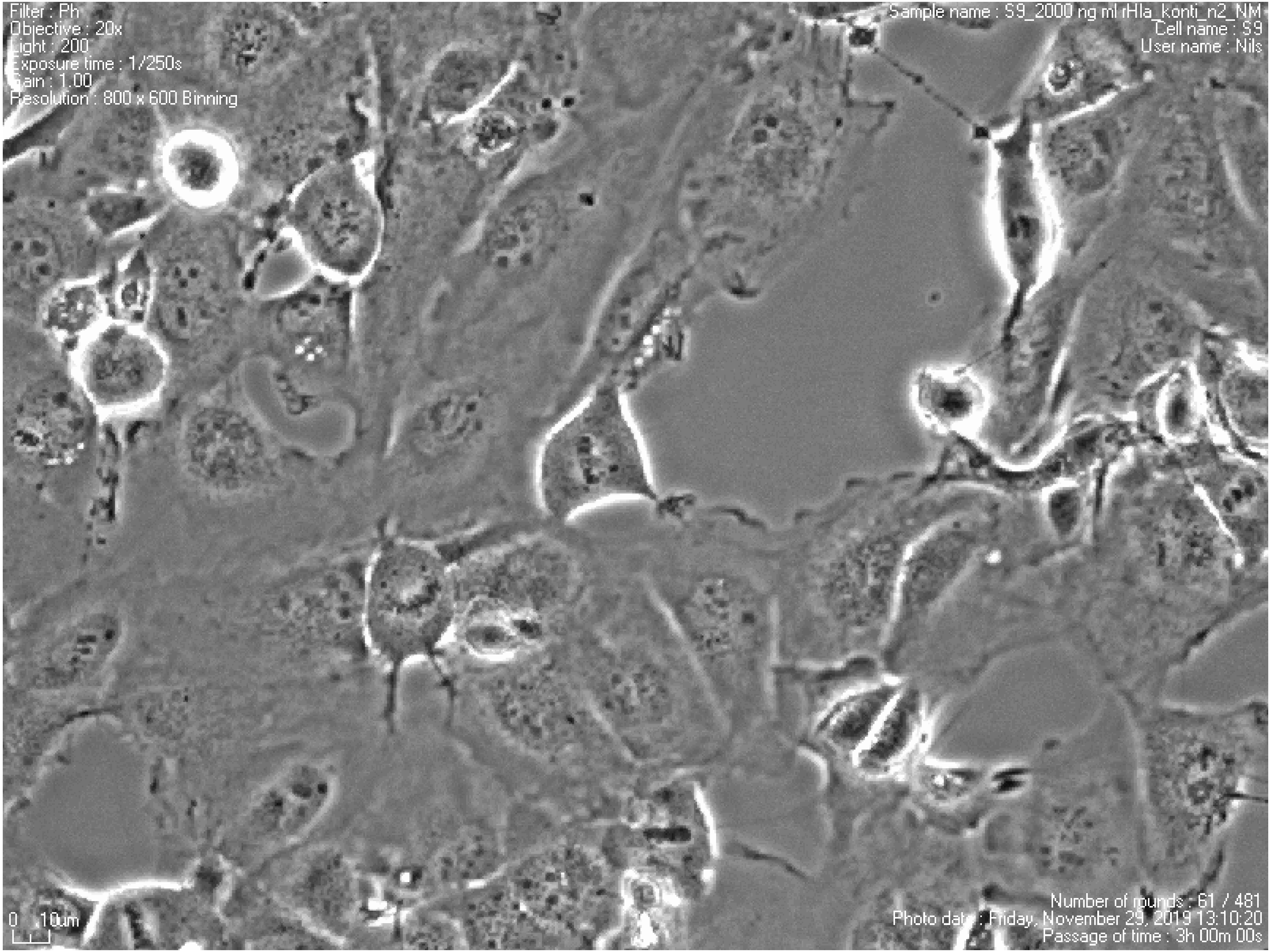
Filter : Ph
Objective : 20x
Light : 200
Exposure time : 1/250s
Gain : 1.00
Resolution : 800 x 600 Binning
Sample name : S9_2000 ng ml rHla_konti_n2_NM
Cell name : S9
User name : Nils
0 10um
Number of rounds : 61 / 481
Photo date : Friday, November 29, 2019 13:10:20
Passage of time : 3h 00m 00s

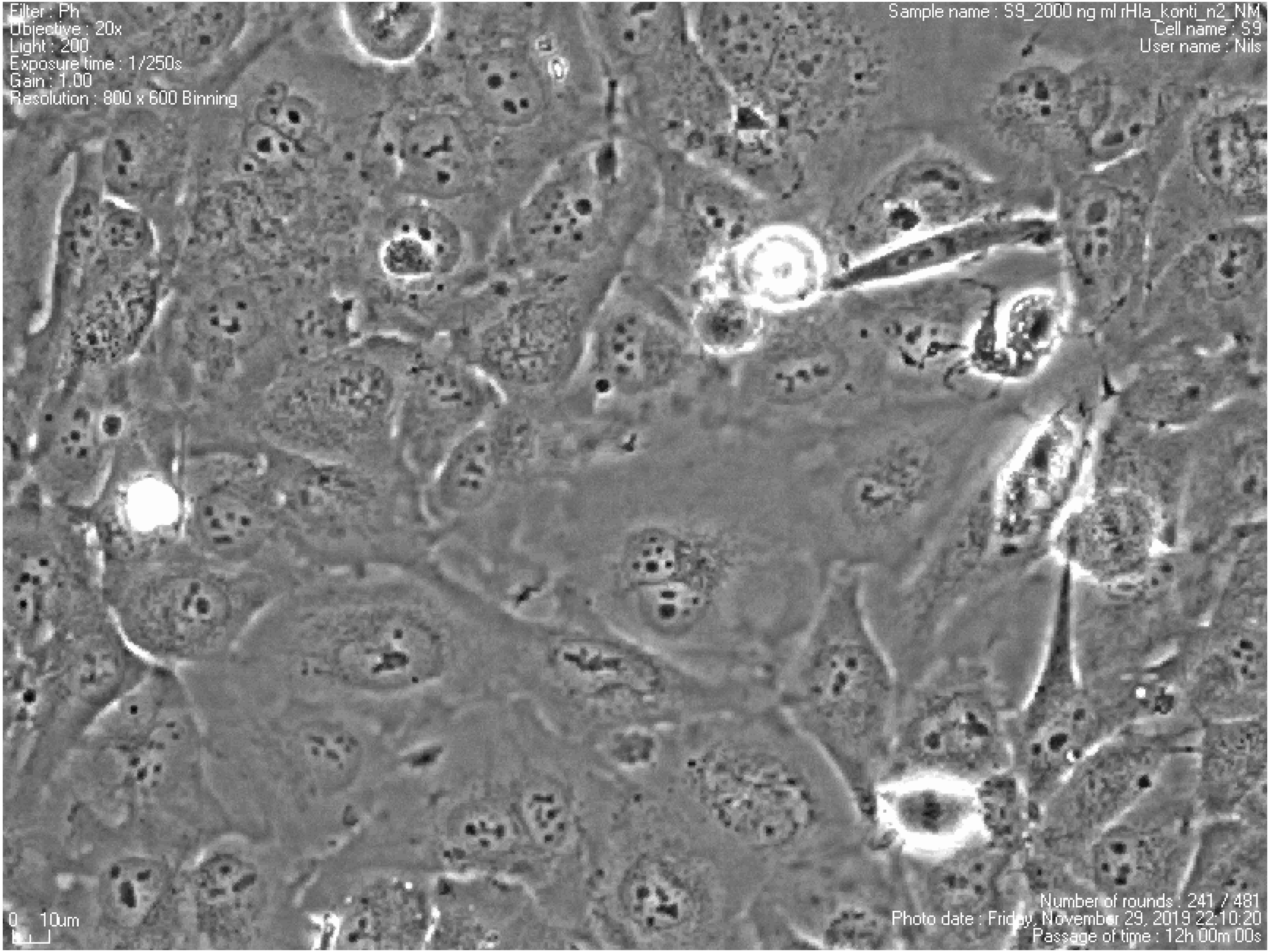
Filter : Ph
Objective : 20x
Light : 200
Exposure time : 1/250s
Gain : 1.00
Resolution : 800 x 600 Binning
Sample name : S9_2000 ng ml rHla_konti_n2_NM
Cell name : S9
User name : Nils
0 10um
Number of rounds : 241 / 481
Photo date : Friday, November 29, 2019 22:10:20
Passage of time : 12h 00m 00s

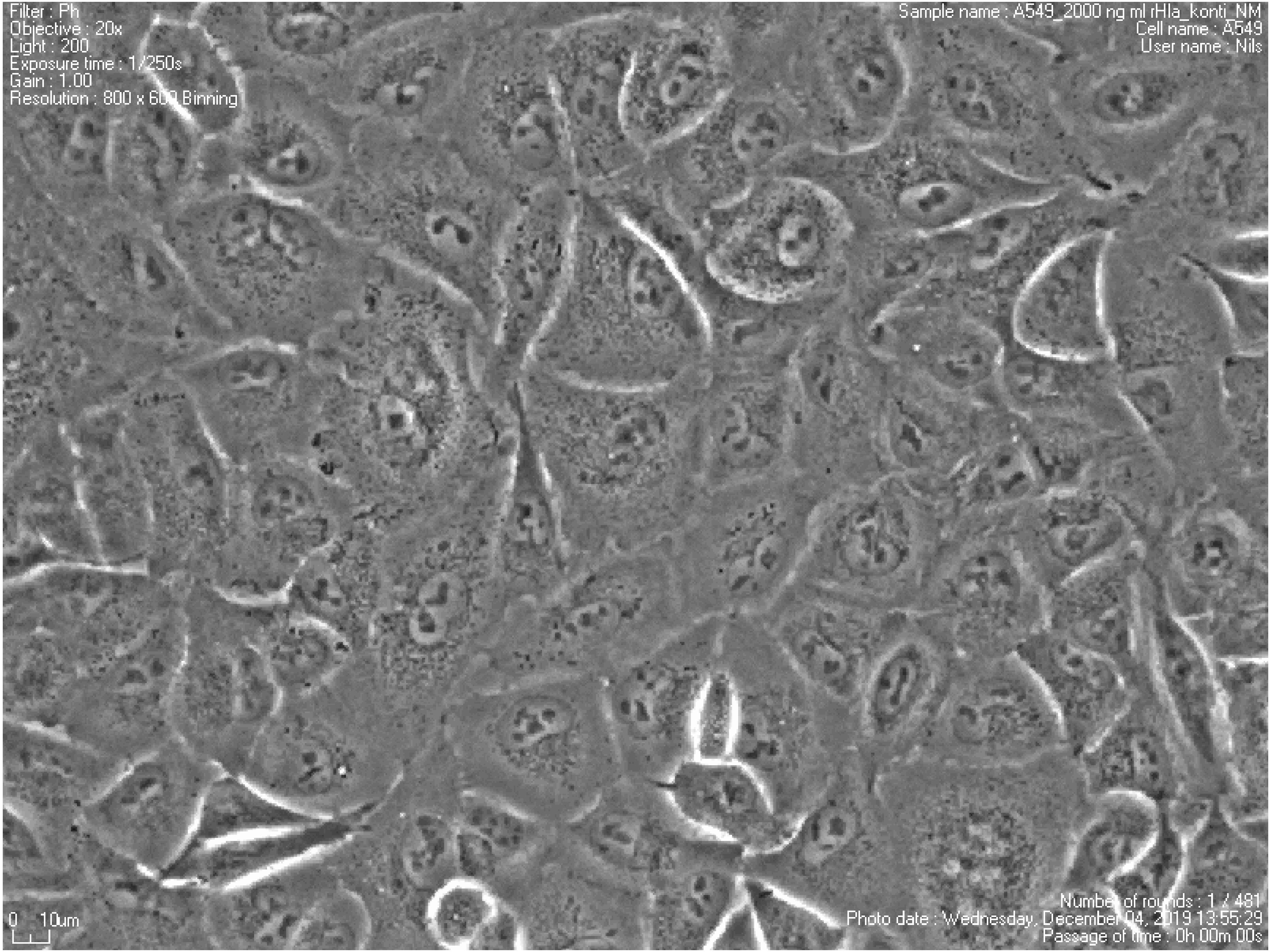
Filter : Ph
Objective : 20x
Light : 200
Exposure time : 1/250s
Gain : 1.00
Resolution : 800 x 600 Binning
Sample name : A549_2000 ng ml rHla_konti_NM
Cell name : A549
User name : Nils
0 10um
Number of rounds : 1 / 481
Photo date : Wednesday, December 04, 2019 13:55:29
Passage of time : 0h 00m 00s

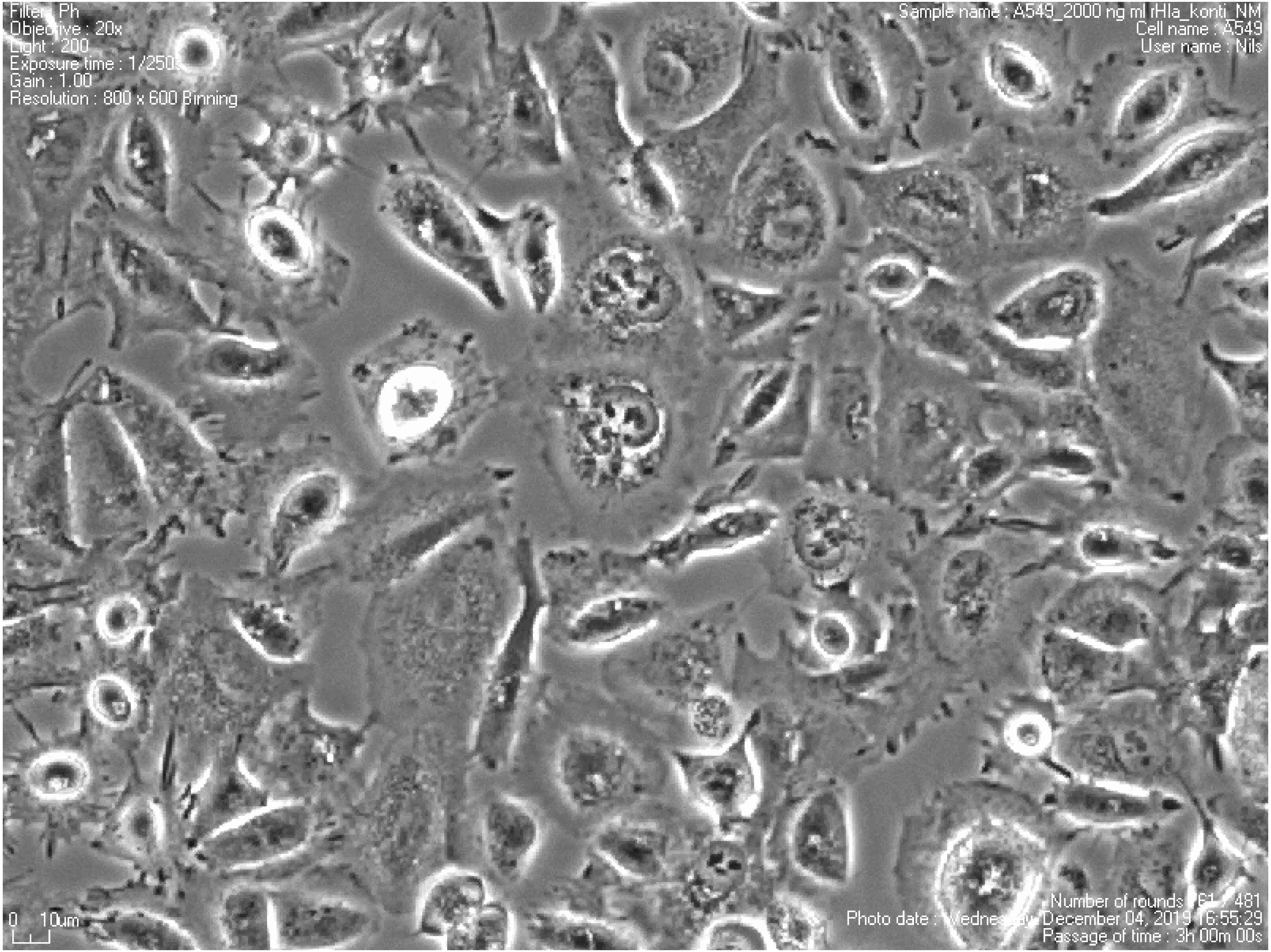
Filter : Ph
Objective : 20x
Light : 200
Exposure time : 1/250s
Gain : 1.00
Resolution : 800 x 600 Binning
Sample name : A549_2000 ng ml rHla_konti_NM
Cell name : A549
User name : Nils
0 10um
Number of rounds : 61 / 481
Photo date : Wednesday December 04, 2019 16:55:29
Passage of time : 3h 00m 00s

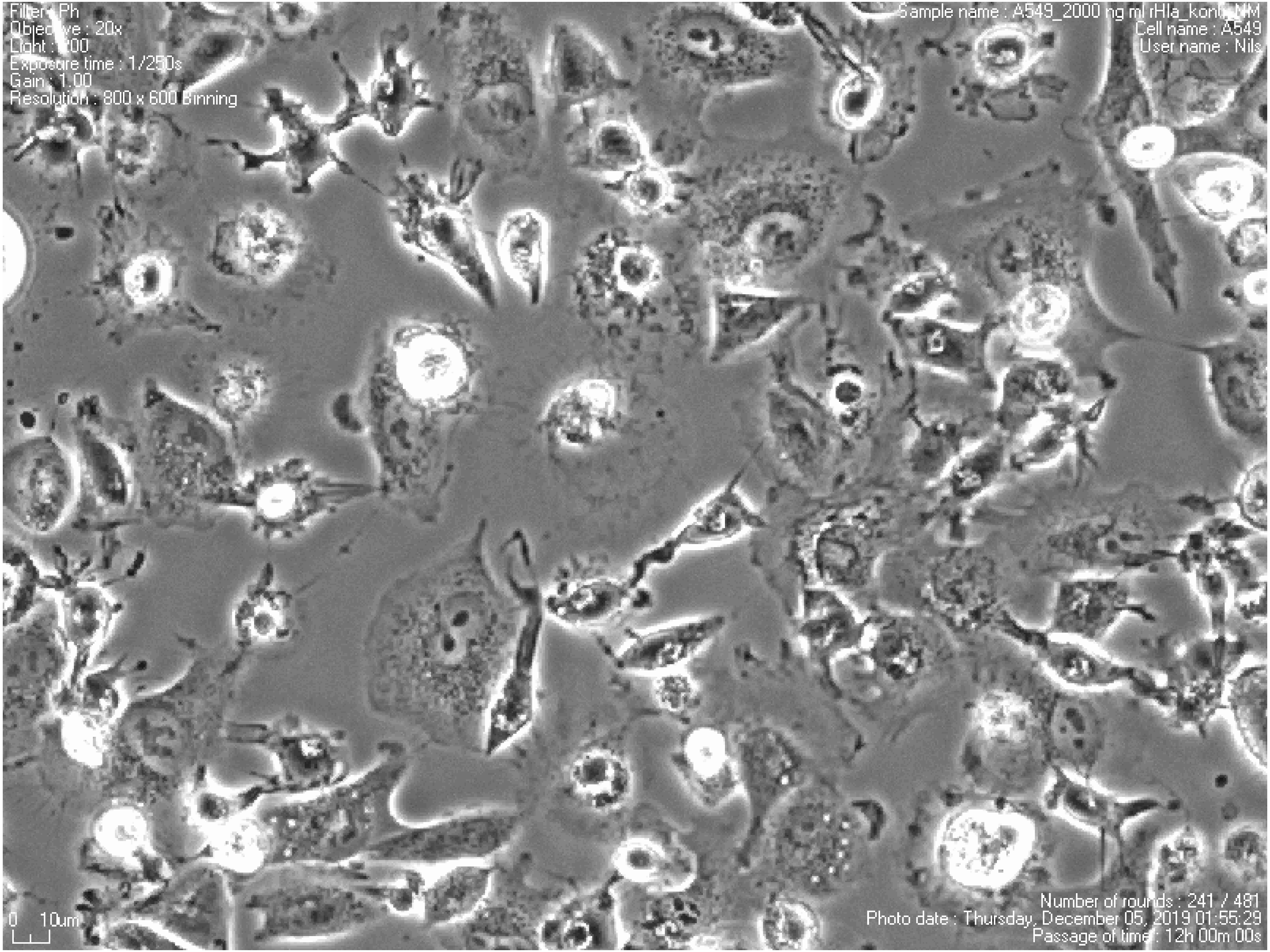
Filter : Ph
Objective : 20x
Exposure time : 1/250s
Gain : 1.00
Resolution : 800 x 600 Binning
Sample name : A549_2000 ng ml rHla_kon
Cell name : A549
User name : Nils
0 10um
Number of rounds : 241 / 481
Photo date : Thursday, December 05, 2019 01:55:29
12h 00m 00s
