# Supplementary figures and images for: S. aureus alpha-toxin monomer binding and heptamer formation in host cell membranes – Do they determine sensitivity of airway epithelial cells toward the toxin?
Source: PLoS One. 2020 May 29;15(5):e0233854. doi: 10.1371/journal.pone.0233854 (PMC7259691; doi:10.1371/journal.pone.0233854)

118 kD

66 kD

43 kD

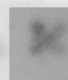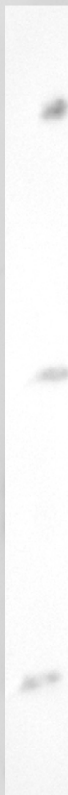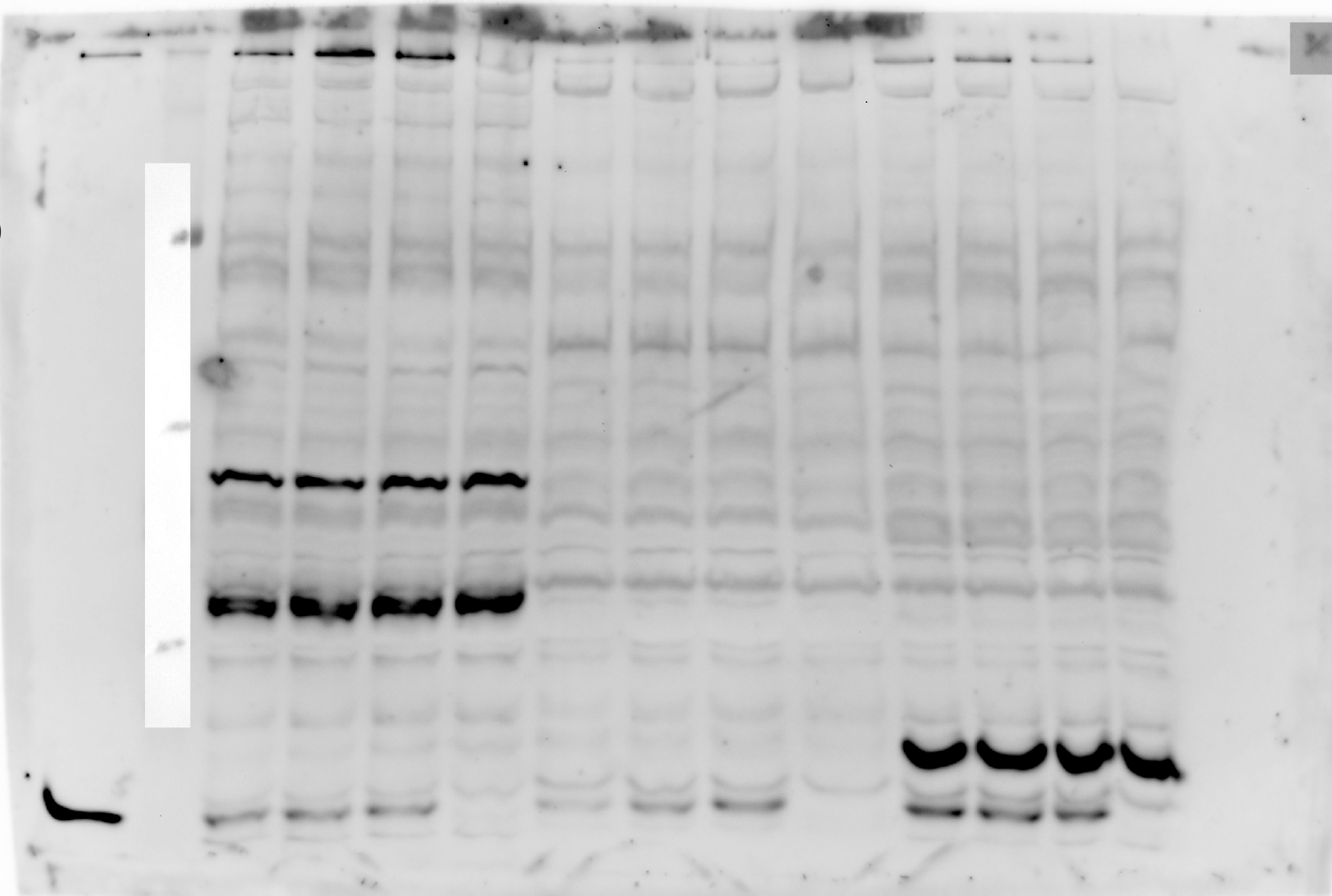

Supplement: S2 Fig — (PDF) [file pone.0233854.s003.pdf]

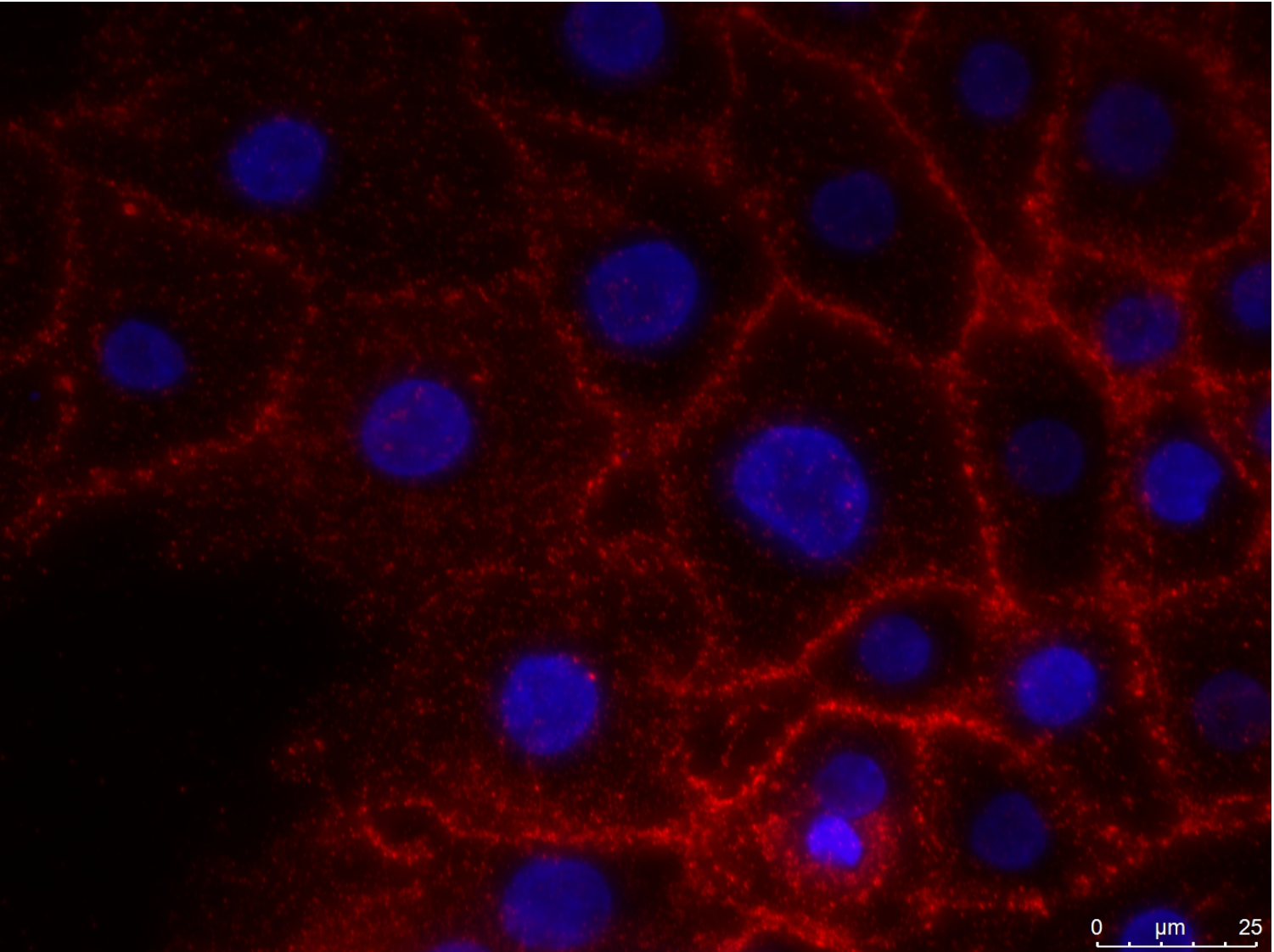

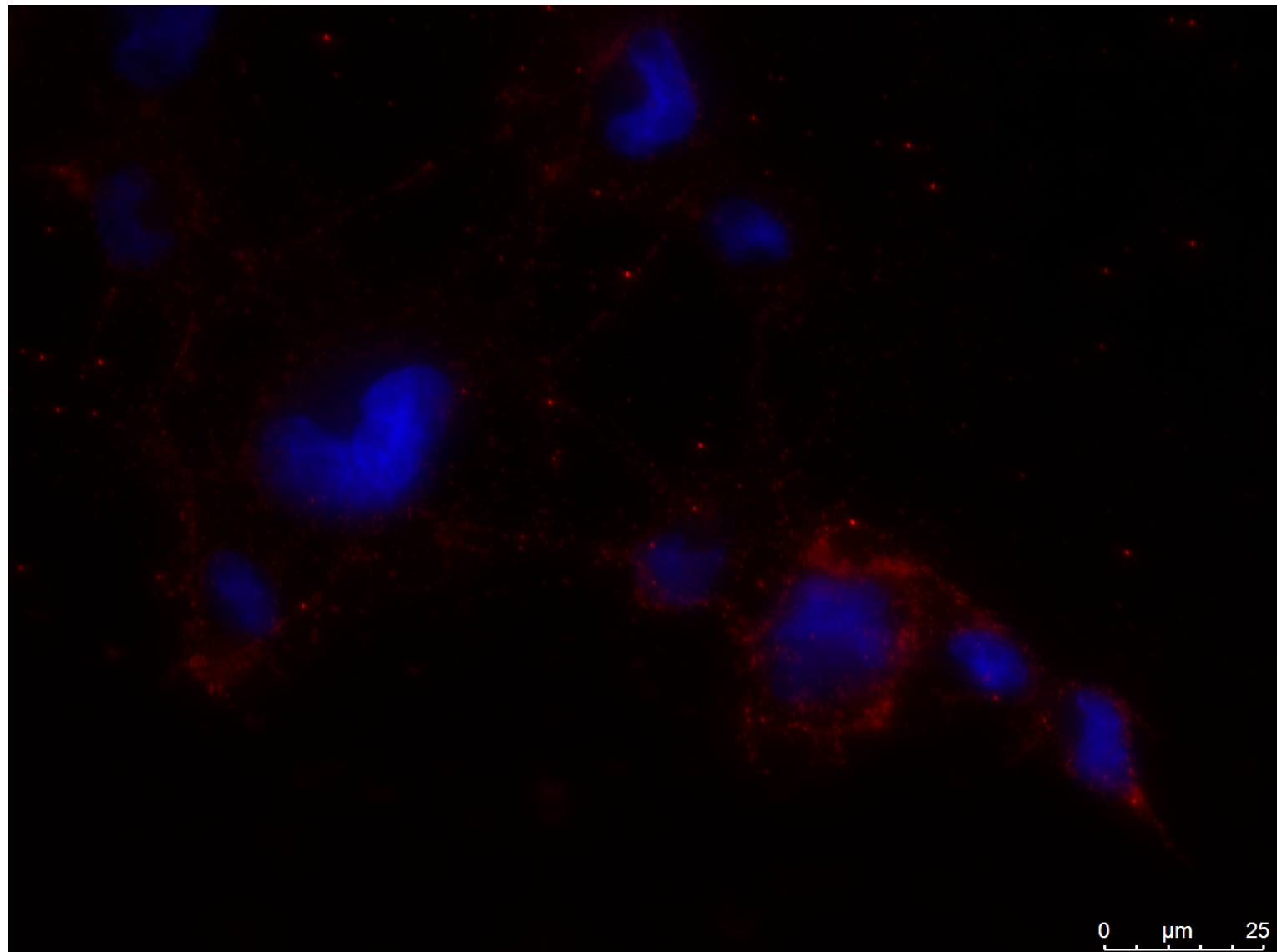

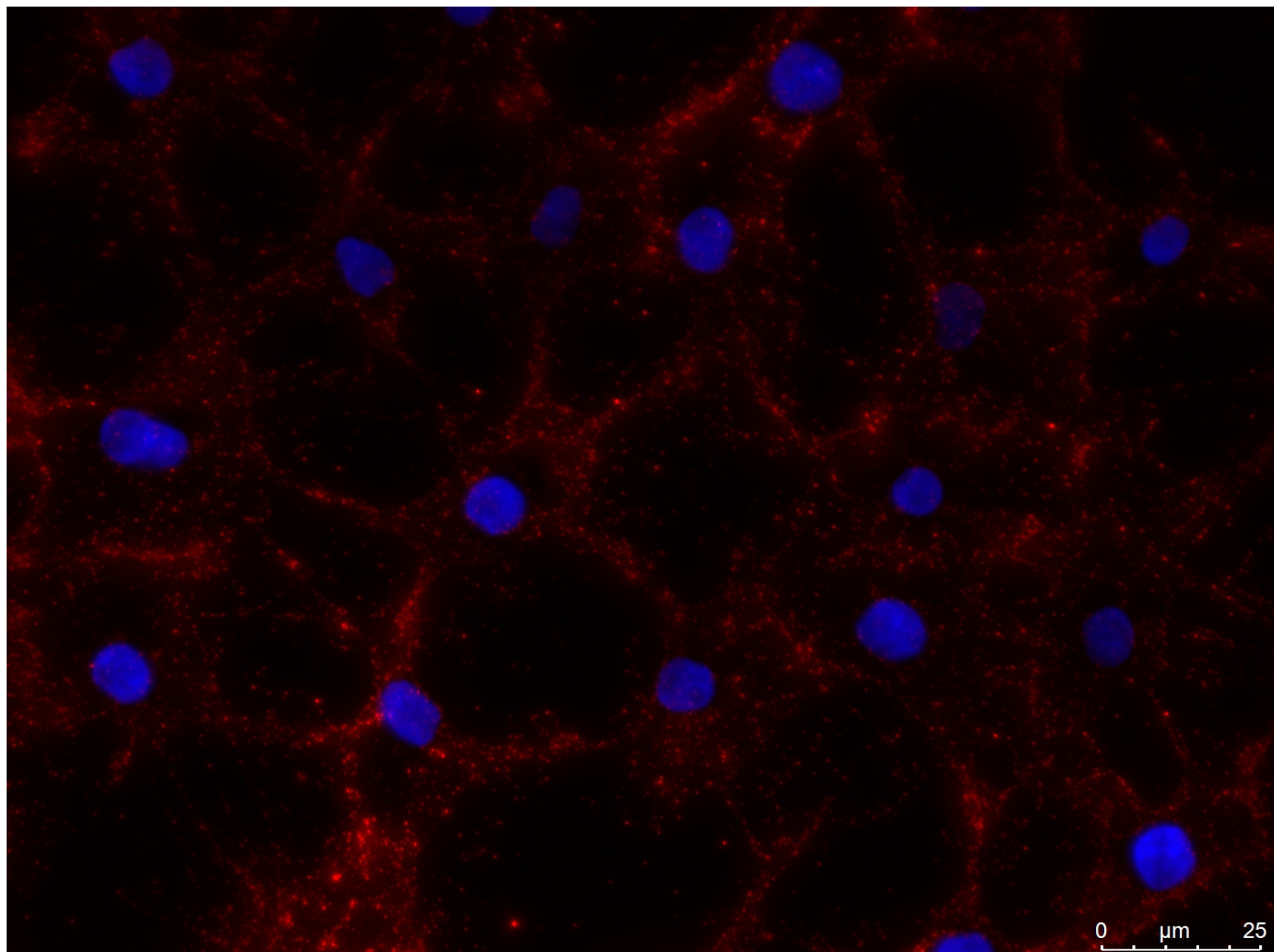

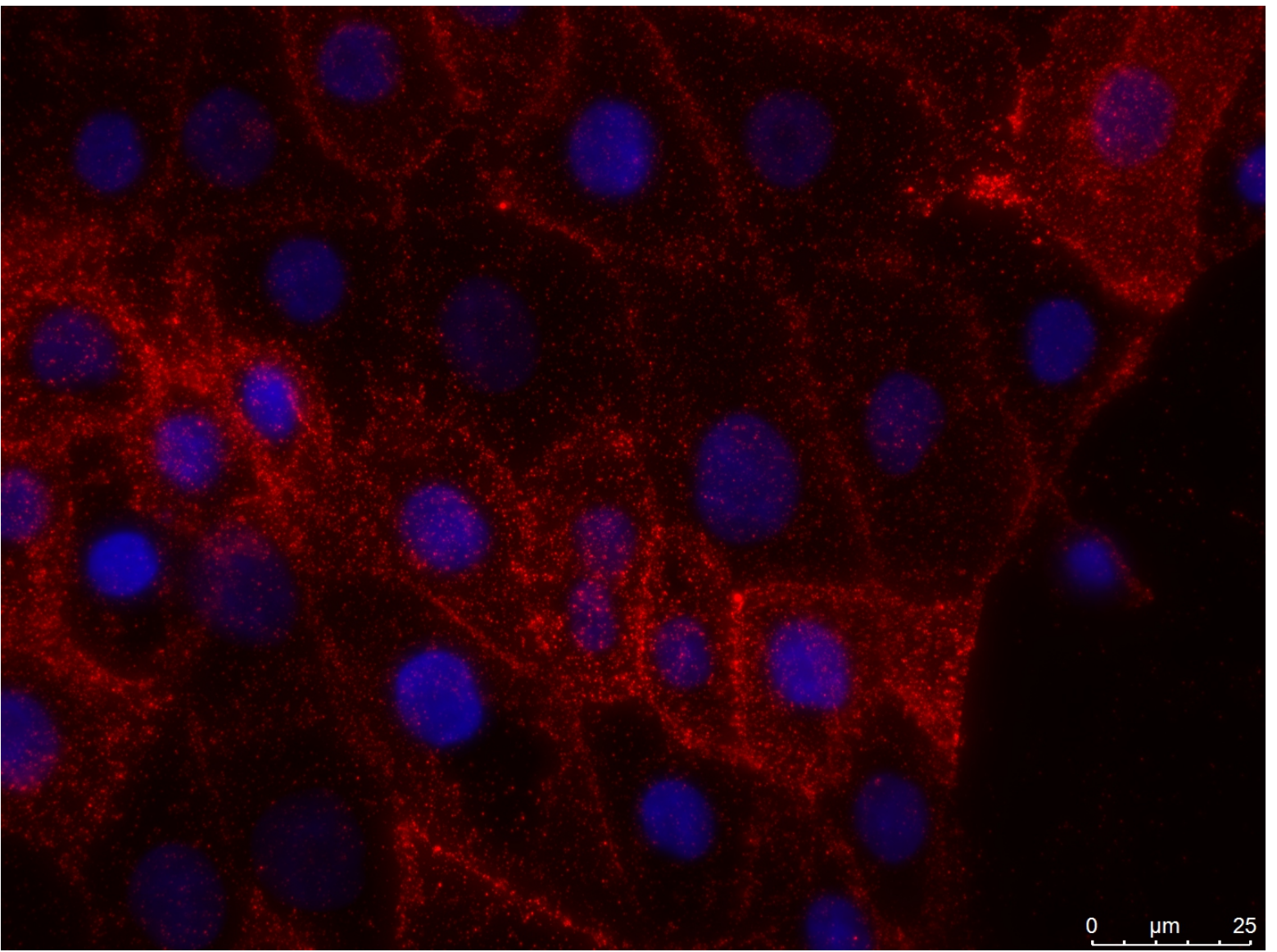

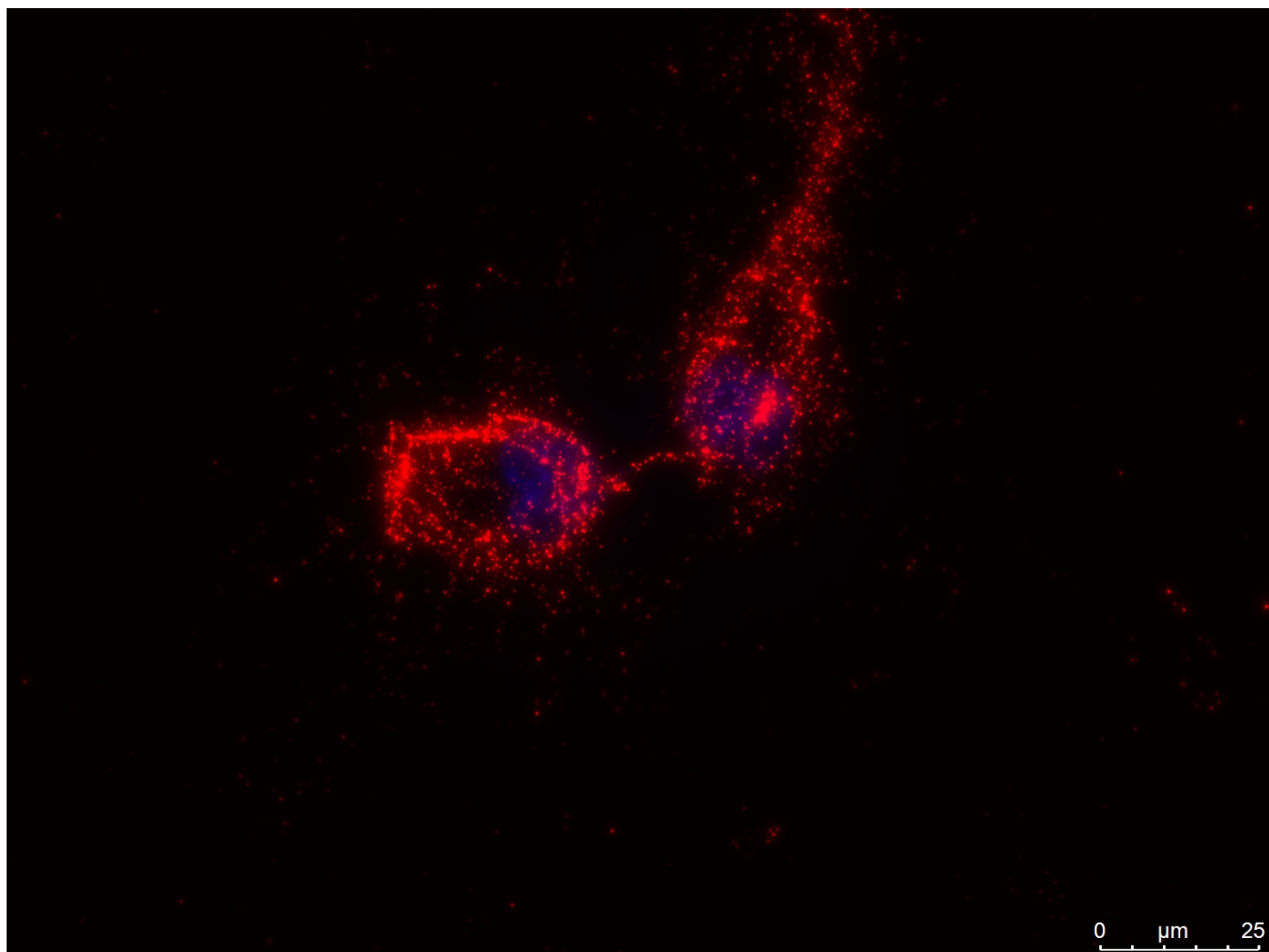

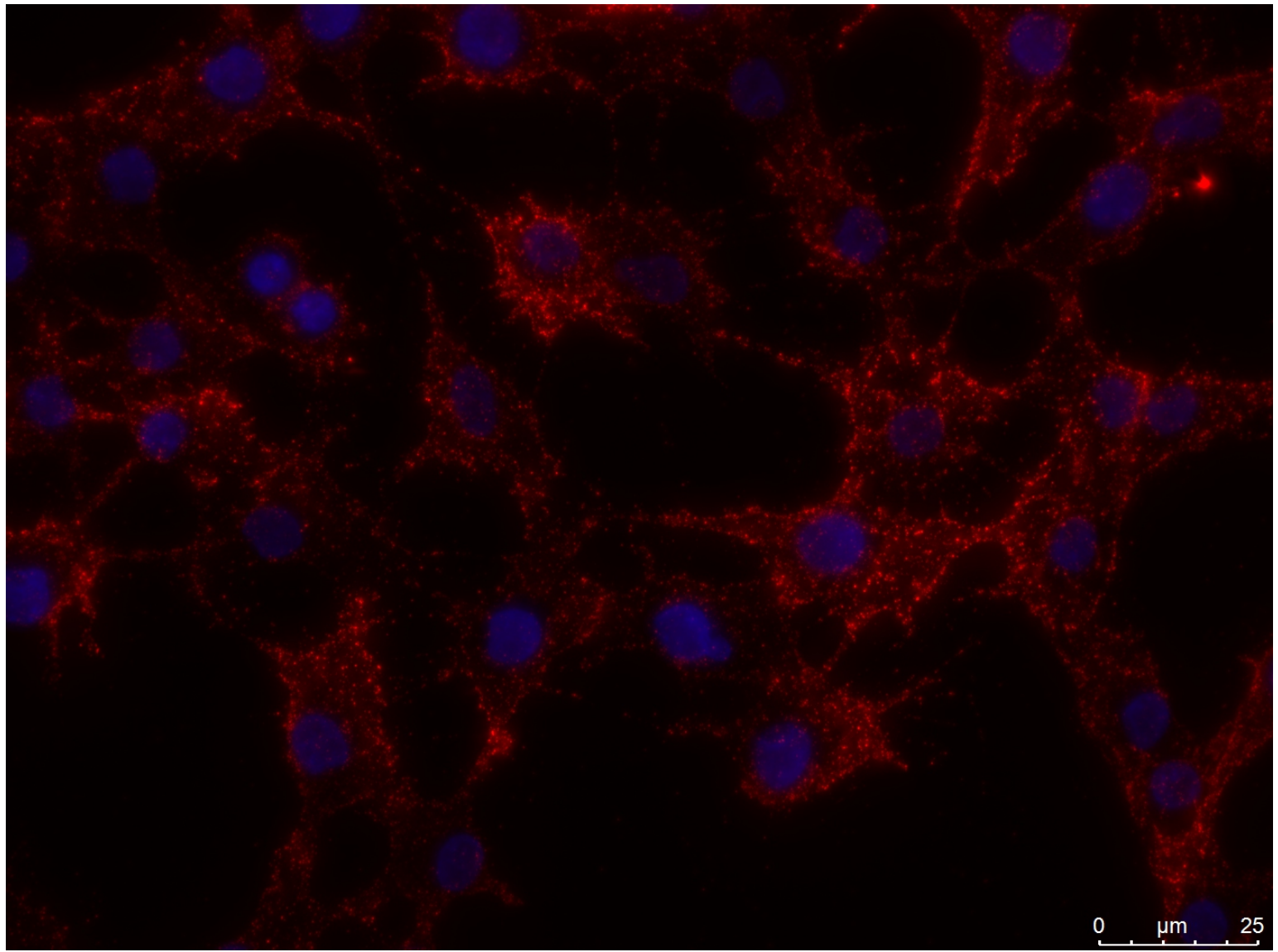

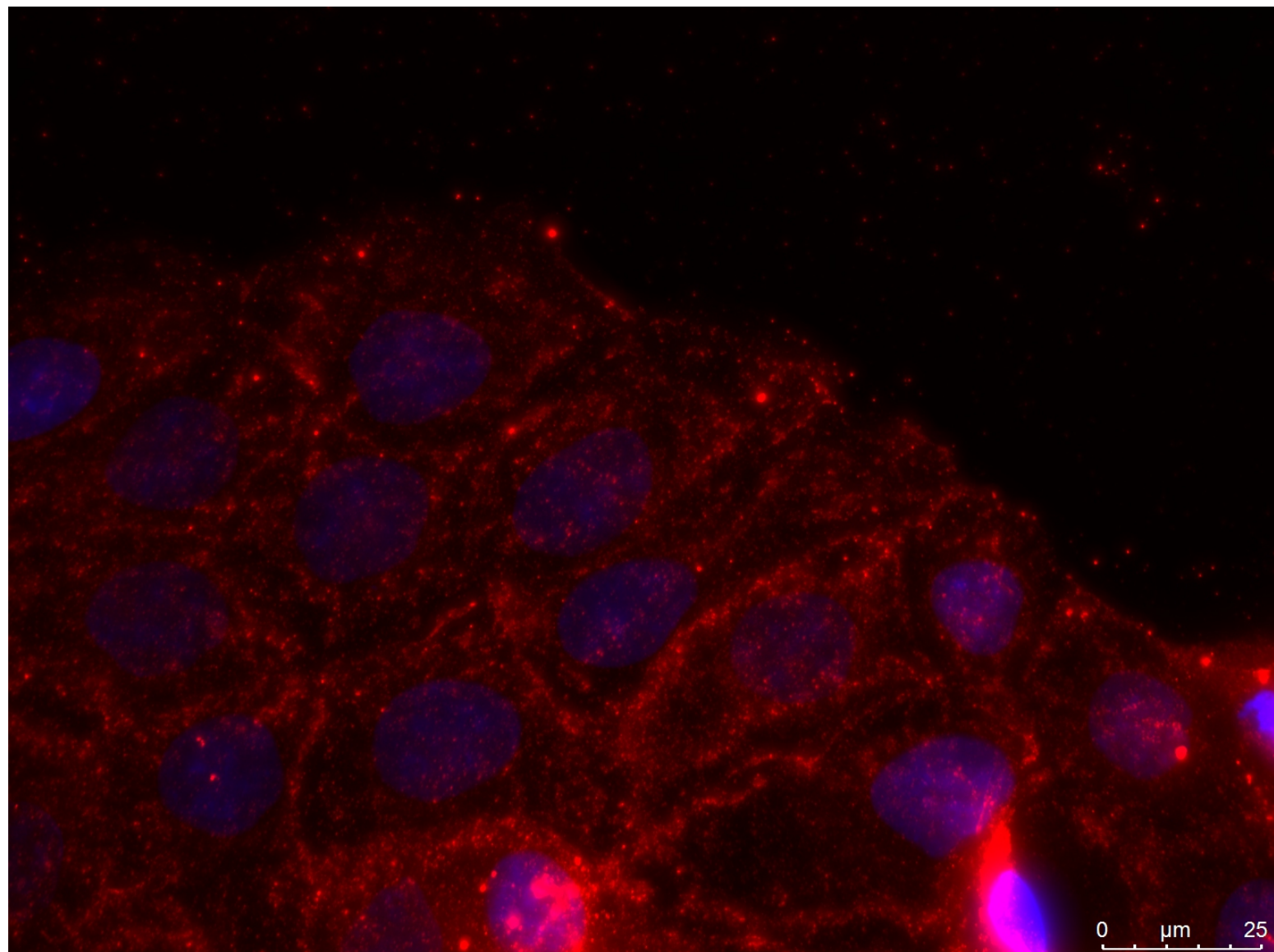

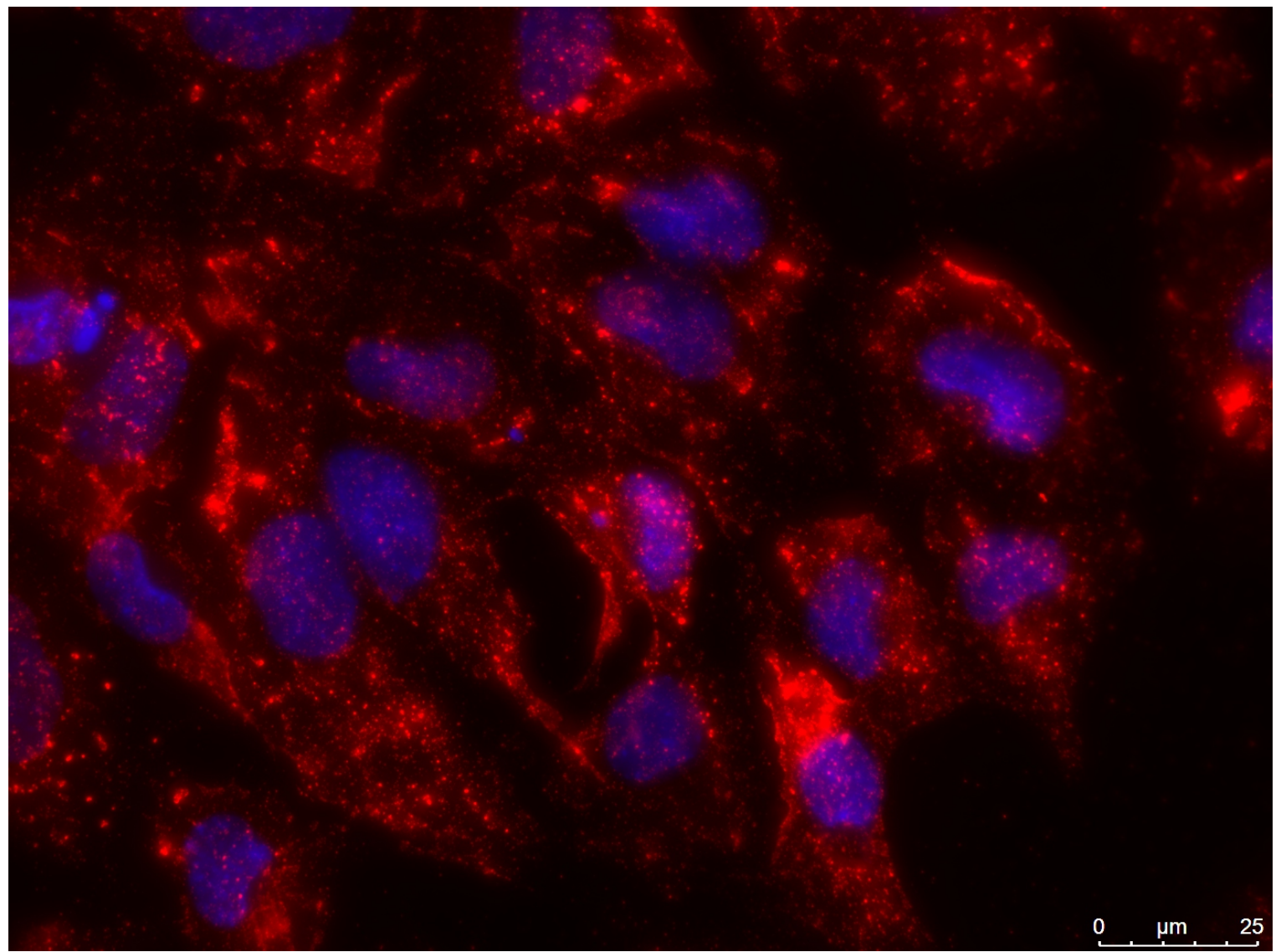

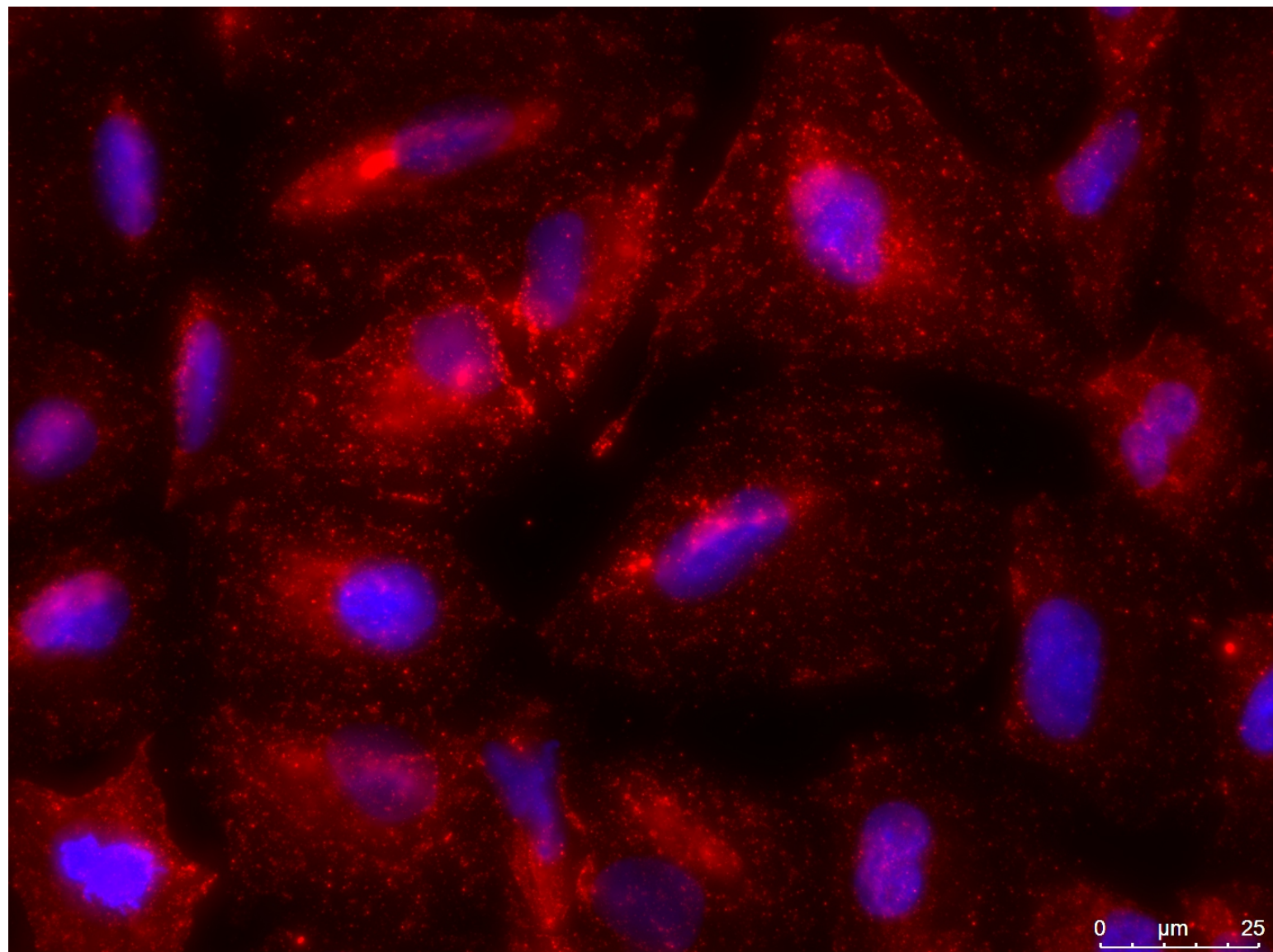

Supplement: S3 Fig — (PDF) [file pone.0233854.s004.pdf]

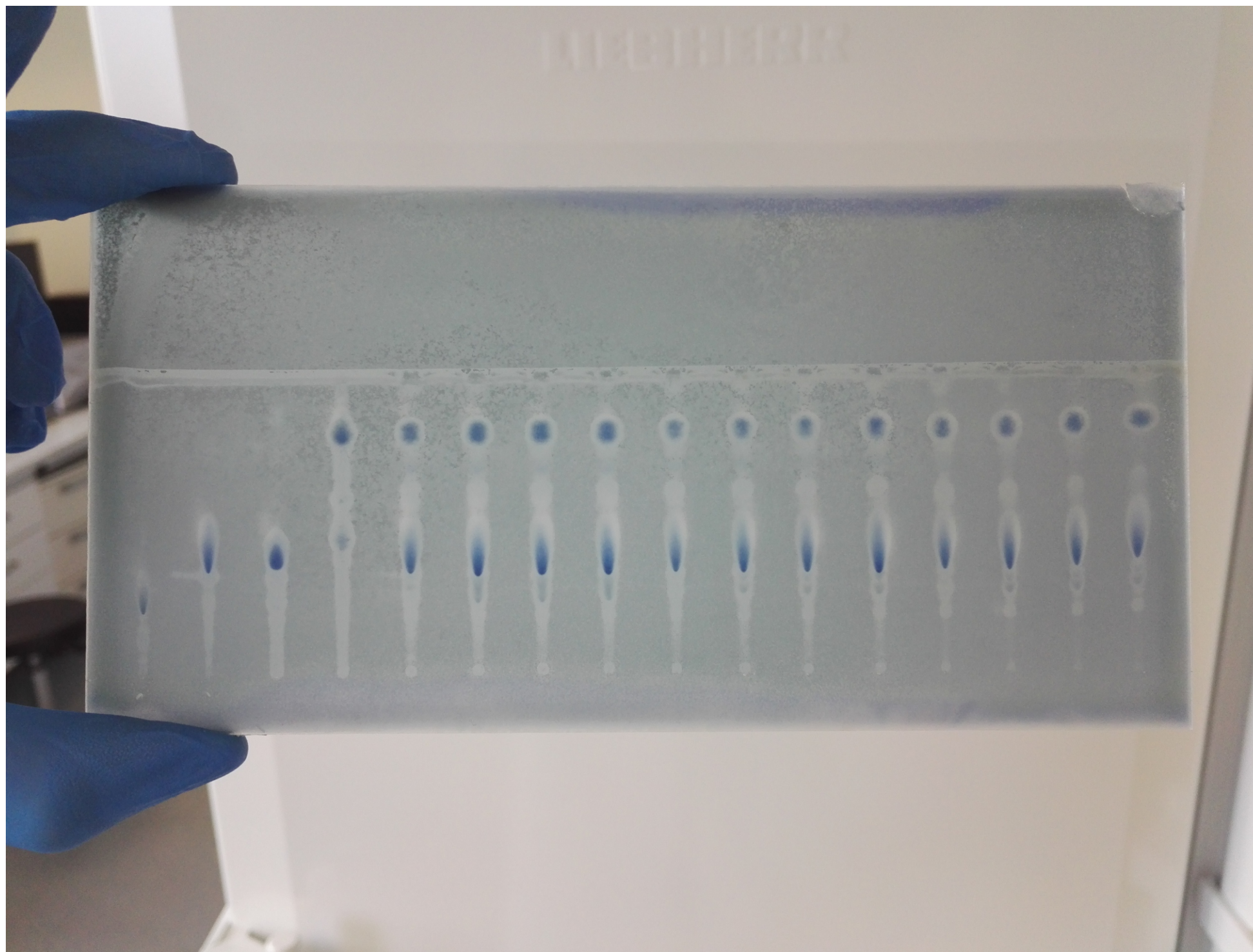

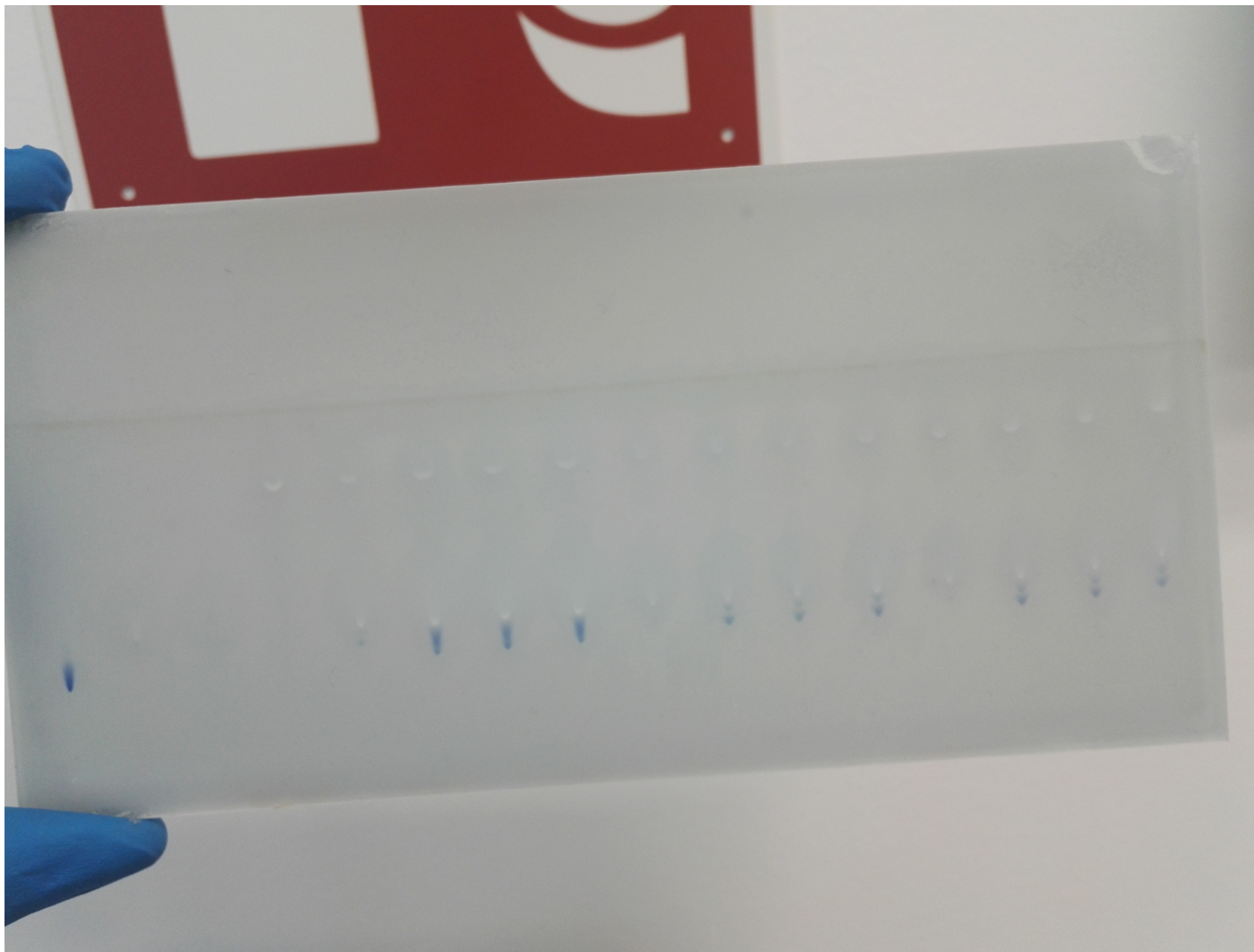

Supplement: S4 Fig — (PDF) [file pone.0233854.s005.pdf]
